# *ARID2* Deficiency Enhances Tumor Progression via ERBB3 Signaling in *TFE3*-Rearranged Renal Cell Carcinoma

**DOI:** 10.3390/cimb46120817

**Published:** 2024-12-02

**Authors:** Jinglong Tang, Shintaro Funasaki, Hidekazu Nishizawa, Shoichiro Kuroda, Takanobu Motoshima, Chang Wu, Amany Sayed Mawas, Yorifumi Satou, Yuichiro Arima, Hisashi Hasumi, Ryosuke Jikuya, Kazuhide Makiyama, Yuichi Oike, Yasuhito Tanaka, Masaya Baba, Tomomi Kamba

**Affiliations:** 1 Department of Urology, Graduate School of Medical Sciences, Kumamoto University, Kumamoto 860-8556, Japan; 213r5151@st.kumamoto-u.ac.jp (J.T.); hidekazu0414@kumamoto-u.ac.jp (H.N.); 117m1035@gmail.com (S.K.); tmoto4ma@yahoo.co.jp (T.M.); kamba@kumamoto-u.ac.jp (T.K.); 2 Division of Molecular and Vascular Biology, IRDA, Kumamoto University, Kumamoto 860-0811, Japan; shintarof@kumamoto-u.ac.jp; 3 Department of Gastroenterology and Hepatology, Graduate School of Medical Sciences, Kumamoto University, Kumamoto 860-8556, Japan; wuchang9712@163.com (C.W.); amany_jaber@vet.svu.edu.eg (A.S.M.); ytanaka@kumamoto-u.ac.jp (Y.T.); 4 Department of Pathology & Clinical Pathology, Faculty of Veterinary Medicine, South Valley University, Qena 83523, Egypt; 5 Division of Genomics and Transcriptomics, Joint Research Center for Human Retrovirus Infection, Kumamoto University, Kumamoto 860-0811, Japan; y-satou@kumamoto-u.ac.jp; 6 Developmental Cardiology Laboratory, International Research Center for Medical Science (IRCMS), Kumamoto University, Kumamoto 860-0811, Japan; arimay@kumamoto-u.ac.jp; 7 Department of Urology, Yokohama City University Graduate School of Medicine, 3-9 Fuku-ura, Kanazawa-ku, Yokohama 236-0004, Japan; hasumi@yokohama-cu.ac.jp (H.H.); jikuya@yokohama-cu.ac.jp (R.J.); makiya@yokohama-cu.ac.jp (K.M.); 8 Department of Molecular Genetics, Graduate School of Medical Sciences, Kumamoto University, Kumamoto 860-8556, Japan; oike@gpo.kumamoto-u.ac.jp

**Keywords:** TFE3-RCC, TFE3 fusion, *ARID2*, ERBB3

## Abstract

*TFE3*-rearranged Renal Cell Carcinoma (TFE3-RCC) is an aggressive subtype of RCC characterized by Xp11.2 rearrangement, leading to TFE3 fusion proteins with oncogenic potential. Despite advances in understanding its molecular biology, effective therapies for advanced cases remain elusive. This study investigates the role of *ARID2*, a component of the SWI/SNF chromatin remodeling complex, in TFE3-RCC. Through a series of in vitro and in vivo experiments, we confirmed that *ARID2* acts as a tumor suppressor in TFE3-RCC. *ARID2* knockout (KO) enhanced TFE3-RCC cell migration, proliferation, and tumor growth. Transcriptomic analysis revealed ERBB3 as a key target gene regulated by both PRCC-TFE3 and *ARID2*. Chromatin immunoprecipitation (ChIP) assays demonstrated that PRCC-TFE3 directly binds to and upregulates ERBB3 expression, with *ARID2* KO further enhancing this effect. TFE3-RCC *ARID2* KO cells exhibited significant gene expression enrichment in MAPK and ERBB3 signaling pathways. These cells also showed increased activation of ERBB3, EGFR, and selective activation of SRC and MAPK. TFE3-RCC *ARID2* KO cells demonstrated heightened sensitivity to the ERBB3 inhibitor AZD8931 compared to their wild-type counterparts, exhibiting significantly reduced migration and proliferation rates. These findings suggest that the PRCC-TFE3-*ARID2*-ERBB3 axis plays a critical role in TFE3-RCC pathogenesis and highlights the potential of targeting ERBB3 in *ARID2*-deficient TFE3-RCC as a therapeutic strategy. This study provides new insights into the molecular mechanisms of TFE3-RCC and suggests avenues for precision treatment of this aggressive cancer.

## 1. Introduction

*TFE3*-rearranged Renal Cell Carcinoma (TFE3-RCC) is a distinct subset of RCC, defined in the 2022 WHO classification, characterized by Xp11.2 rearrangements that result in *TFE3* gene fusions with various partner genes [1,2,3]. TFE3-RCC typically affects children and younger patients; however, it can also occur in middle-aged and older adults [4,5]. It often presents with aggressive features and a poor prognosis, with no standard effective therapy for advanced cases, highlighting the urgent need for a deeper understanding of its molecular pathogenesis and the development of targeted therapeutic strategies [6,7,8]. TFE3 is a transcription factor featuring a bHLH-Zip structure essential for dimerization and DNA binding. In TFE3-RCC, all identified *TFE3* fusion genes encode chimeric proteins that preserve this critical bHLH-Zip domain [3,6,8,9]. These chimeric TFE3 proteins are thought to function as oncogenic drivers, exhibiting constitutive transcriptional activity [8,10,11,12,13,14]. Indeed, we have previously demonstrated that the expression of a chimeric TFE3, PRCC-TFE3, in mouse kidney epithelial cells leads to the development of RCC, proving the oncogenic function of PRCC-TFE3. Notably, this mouse model exhibits a variety of histologies in the kidney, including cysts, hyperplasia, adenoma, and RCC [8]. This histological heterogeneity suggests that PRCC-TFE3 may cooperate with additional genetic or epigenetic events in the development and progression of TFE3-RCC [8]. Recent genomic profiling studies have revealed a landscape of genetic alterations in TFE3-RCC, in addition to the *TFE3* rearrangement [6,15,16]. However, none of the candidate driver genes have been functionally proven yet. Among the candidate genes identified, we have been particularly interested in mutations of AT-Rich Interaction Domain 2 (*ARID2*) and *SMARCA4*. Both of these genes encode components of the SWI/SNF chromatin remodeling complexes, which are implicated in various cancers, suggesting their potential importance in TFE3-RCC as well [17,18,19,20,21,22]. The SWI/SNF multi-subunit protein complex utilizes energy derived from ATP hydrolysis to remodel the chromatin structure, thereby regulating DNA accessibility for various cellular processes such as transcription, replication, and DNA repair [23,24]. This complex is composed of two distinct subtypes: BAF (BRG1/BRM-associated factor) and PBAF (polybromo-associated BAF) [25,26,27,28]. Mutations in the BAF or PBAF components are associated with various cancers, indicating their tumor suppressor function. *PBRM1*, a component of the PBAF complex, is highly mutated in approximately 40% of clear cell renal cell carcinoma (ccRCC) cases [29,30,31,32,33]. *PBRM1* mutation is also found in many cancers including cholangiocarcinoma, lung adenocarcinoma (LUAD), bladder urothelial carcinoma (BLCA), skin cutaneous melanoma (SKCM), and non-small cell lung cancer (NSCLC) [30,34,35,36]. Another component of the PBAF complex, *ARID2*, is also frequently mutated in various types of cancers. Notably, *ARID2* mutations are prevalent in hepatocellular carcinoma (HCC), melanoma, and NSCLC [18,37,38]. Of particular interest are *ARID2* mutations in melanoma [37,39]. This is because microphthalmia-associated transcription factor (MITF), a member of the MiT/TFE family of transcription factors that includes TFE3, serves as a master regulator of melanocyte biology and acts as a lineage survival oncogene in melanoma [40,41]. *ARID2* mutations in melanoma may alter chromatin accessibility, potentially leading to abnormal transcription by MITF and consequently contributing to melanoma development and progression [23]. In addition, the natural mutations of *ARID2* further highlight its importance as a tumor suppressor in TFE3-RCC [16]. Therefore, we hypothesized that the loss of *ARID2* function may synergize with the oncogenic fusion TFE3, contributing to the progression of TFE3-RCC. This study aims to elucidate the functional consequences of *ARID2* deficiency in TFE3-RCC, investigating its impact on tumor biology, gene expression patterns, molecular mechanism, and potential therapeutic targets.

## 2. Materials and Methods

### 2.1. Cell Culture

The cell line used in this study included the renal cell carcinoma cell line UOK124 (gifts of Dr. W. Marston Linehan, National Cancer Institute, Bethesda, MD, USA). UOK124 cells and 293LTV cells were cultured in DMEM (Gibco, Grand Island, NY, USA) supplemented with 10% FBS (Gibco) and 1% penicillin/streptomycin (Invitrogen, Carlsbad, CA, USA). HK-2 cell lines which express HA-PRCC-TFE3 in a doxycycline-dependent manner were established using the Tet-On^®^ 3G Inducible Expression System and cultured in Advanced DMEM/F-12 with 1.5% Tetracycline-Free Fetal Bovine Serum and selection antibiotics, 2 mg/mL Blasticidin S and 0.8 mg/mL Puromycin.

### 2.2. Plasmids, Lentiviral Vector Production, and Cell Transfection

The LentiCRISPRv2 plasmid (Addgene #52961) was employed to generate *ARID2* KO in UOK124 cells. Initially, the LentiCRISPRv2 plasmid was subjected to BsmBI restriction enzyme digestion, followed by gel purification. Subsequently, *ARID2*-specific sgRNA sequences were designed and synthesized (sgRNA Sense #1: 5′-CACC GCCCTTTCTCCGCTCGTCCGG, sgRNA Antisense #1: 5′-AAACCCGGACGAGCGG AGAAAGGGC). These sgRNA oligonucleotides were then cloned into the BsmBI- digested LentiCRISPR v2 plasmid. The resulting constructs were verified through DNA sequencing analysis. Twenty-four hours after seeding 1 × 10^6^ 293LTV cells in a six-well plate, the cells were transfected with LentiCRISPR v2-*ARID2* along with packaging vectors using PEI as the transfection reagent. The culture medium was replaced 24 h post-transfection. Subsequently, the supernatant was harvested 48 h after the initial transfection. The supernatant was filtered through a 0.45 μm PVDF membrane to remove cellular debris and subsequently stored for future use. Concurrently, UOK124 cells were seeded in 10 cm culture dishes at a density of 5.0 × 10^6^ cells per dish. To transduce the cells, 2 mL of viral supernatant and polybrene at a concentration of 5 μg/mL were added to each dish. Forty-eight hours post-transduction, puromycin was introduced to the culture medium at a final concentration of 5 μg/mL to select for successfully transduced cells. This selection process was maintained for an additional 2–3 days.

### 2.3. Single-Cell Clones Isolation

Puromycin-selected cells were diluted to 0.5 cells/well in 96-well plates using limiting dilution. Single colonies were identified microscopically after 3–5 days. These were expanded sequentially in 24-well and 6-well plates over two weeks. *ARID2* KO in individual clones was verified by Western blotting of total protein extracts from confluent cultures.

### 2.4. Antibodies

The antibodies utilized in this study are detailed in Table 1.

### 2.5. Cell Proliferation Assay

UOK124 WT and *ARID2* KO cells were cultured to near confluence, then harvested using Acutase and resuspended in fresh medium. Cells were quantified and seeded at a density of 2 × 10^4^ cells per well in 6-well plates, with triplicate wells for each experimental condition. The study included groups treated with and without the AZD8931 inhibitor at a concentration of 20 ng/mL. Cell proliferation was assessed by performing cell counts on days 1, 3, 5, and 7 using a Bio-Rad TC20 automated cell counter (Hercules, CA, USA). Growth curves were subsequently generated based on these measurements.

### 2.6. Cell Scratch Assay

UOK124 WT and *ARID2* KO cells were seeded at a density of 2 × 10^4^ cells per well in 6-well plates, with triplicate wells for each experimental condition. Cultures were maintained at 37 °C. For the wound healing assay, UOK124 WT and *ARID2* KO cells were treated with or without the AZD8931 inhibitor (20 ng/mL). Upon reaching confluence, a scratch was made using a sterile pipette tip to create a wound. The cells were then washed with PBS, and the medium was replaced with DMEM containing 2% serum. Wound healing progression was monitored using a Keyence BZ X800 imaging system (Osaka, Japan) (4X objective) at 0, 12, 24, 36, and 48 h post-wounding.

### 2.7. Plate Colony Formation Assay

UOK124 WT and *ARID2* KO cells in the logarithmic growth phase were harvested using Accutase (Nakarai, Kyoto, Japan), resuspended, and quantified. The cells were then seeded at a density of 200 cells per well in 6-well plates. The plates were incubated at 37 °C in a 5% CO_2_ atmosphere for 2–3 weeks, with culture medium replenished every three days, until visible colonies formed. Upon colony formation, the cells were washed with PBS and fixed with 4% paraformaldehyde for 30 min. The colonies were then stained with 0.1% crystal violet solution for 30 min. After thorough rinsing and air-drying, the stained colonies were imaged using the Keyence BZ X800 imaging system.

### 2.8. Nude Mice Tumorigenicity Assay

Five-week-old BALB/c-nu/nu mice were utilized for the tumorigenicity assay. UOK124 WT and *ARID2* KO cells were cultured to 80–90% confluence, then harvested using Accutase. The cells were resuspended in a 1:1 mixture of PBS and Matrigel at a concentration of 4 × 10^6^ cells per 100 μL. Following ethanol disinfection of the injection sites, 100 μL of the cell suspension was injected subcutaneously into the bilateral flank areas of each mouse. Tumor growth was monitored at three-day intervals. Tumor volume was calculated using the formula V = 1/2(L × W × H), where L, W, and H represent length, width, and height, respectively. When tumors reached approximately 20 mm in diameter, final measurements were recorded. Subsequently, mice were euthanized using isoflurane anesthesia followed by cervical dislocation. Tumors were then excised and weighed.

### 2.9. RNA-Sequencing

Total RNA was extracted using TRIzol reagent, and its quality was assessed using a BioAnalyzer 2100 (Agilent Technologies, Santa Clara, CA, USA), with samples having an RNA Integrity Number (RIN) greater than 8 being selected for further processing. Library preparation was performed using the TruSeq Stranded mRNA Library Prep kit, following the manufacturer’s protocol. Sequencing was conducted on an Illumina NextSeq 500 platform using the NextSeq 500/550 High Output v2.5 Kit (Illumina, San Diego, CA, USA), generating single-end reads of 75 nucleotides in length. The resulting sequencing reads underwent initial processing using Trim Galore! (version 0.6.6, incorporating cutadapt version 2.5) to remove adapter sequences and trim low-quality ends. The trimmed reads were subsequently aligned to the human genome (UCSC hg38 assembly) using STAR (version 2.7.9a). Transcript quantification and differential expression analysis were performed using a combination of RSEM (version 1.3.3) for Transcripts Per Million (TPM) calculations and DESeq2 (version 1.42.1) for statistical analysis of differential expression. The gene transfer format (GTF) file used for annotation was obtained from the UCSC hg38 genome build.

### 2.10. Quantitative RT-PCR

Total RNA was extracted from UOK124 wild-type and *ARID2* KO cells using TRIzol reagent (Invitrogen, Carlsbad, CA, USA) following the manufacturer’s protocol. The extracted RNA was then reverse-transcribed into cDNA using ReverTra Ace^®^ qPCR RT Master Mix with gDNA Remover (TOYOBO, FSQ-301) (Osaka, Japan). Quantitative PCR (qPCR) was performed using THUNDERBIRD^®^ SYBR™ qPCR Mix (TOYOBO, QPS-201, Osaka, Japan) according to the manufacturer’s instructions. The qPCR reactions were carried out on a LightCycler 96 system (Roche Diagnostics, Basel, Switzerland), with each sample analyzed in triplicate. Gene expression levels were quantified using the ΔΔCt method. The ribosomal protein S18 (Rps18) gene was used as an endogenous reference for normalization. The specific primers used for qPCR are listed in Table 2.

### 2.11. Gene Knockdown

*PRCC-TFE3* knockdown was performed using the miRE-based RNAi system (Addgene, Watertown, MA, USA). The procedure involved cloning shRNA-miRE sequences into SGEP plasmid vectors (Addgene #111170). Subsequently, TFE3-RCC cell lines were transduced with the resulting viral supernatants, followed by selection using puromycin (Promega, Fitchburg, WI, USA). For the construction of miRE, two target sequences were employed: a control sequence (5′-TATTATTTTAATCACAAACCTA-3′) and a shPRCC-TFE3 sequence are as follows.

shTFE3#1 5-TCAGATAAACAAATGAGGGGGT-3;

shTFE3#3 5-TATTATTTTAATCACAAACCTA-3.

### 2.12. ChIP Sequencing Analyses

Doxycycline-inducible HA-TFE3 HK-2 cells were exposed to 200 ng/mL doxycycline for 24 h. The cells were then subjected to cross-linking using 1% formaldehyde for 5 min at room temperature, followed by a quenching step with 125 mM glycine. Subsequent steps, including nuclear extraction, chromatin fragmentation via micrococcal nuclease, immunoprecipitation using anti-HA antibody (3F10, Roche, Basel, Switzerland), and DNA isolation, were carried out using the SimpleChIP Plus Enzymatic Chromatin IP Kit (Cell Signaling Technology, Beverly, MA, USA) as per the manufacturer’s instructions. For next-generation sequencing, DNA libraries were constructed from the immunoprecipitated DNA using NEBNext Ultra DNA Library Prep Kit and NEBNext Multiplex Oligos for Illumina (New England BioLabs, Ipswich, MA, USA). These multiplexed libraries underwent cluster generation and sequencing on the NextSeq desktop platform (Illumina) using a NextSeq 500 Kit (75 cycles) (Illumina, San Diego, CA, USA). The MACS algorithm, implemented in Strand NGS software (Strand Life Sciences, Bengaluru, India), was employed for peak detection. HA-TFE3 binding sites were determined based on statistically significant enrichment of ChIP-seq signals compared to input DNA peaks, using a stringent *p*-value threshold of 5–10. The resulting ChIP-seq data were visualized and analyzed using the StrandNGS 3.4 software.

### 2.13. Chromatin Immunoprecipitation (ChIP) and Quantitative PCR Analysis

UOK124 WT, *ARID2* KO, TFE3 vector, and shTFE3 cells were subjected to chromatin immunoprecipitation. Cells were cross-linked using 1% formaldehyde for 5 min at room temperature, followed by quenching with 125 mM glycine. Subsequent steps, including nuclear extraction, chromatin fragmentation via micrococcal nuclease digestion, immunoprecipitation using anti-TFE3 antibody (#81744, Rabbit mAb, CST) or IgG control (#2729, Rabbit mAb, CST), and DNA isolation, were performed using the SimpleChIP Plus Enzymatic Chromatin IP Kit (Cell Signaling Technology, Beverly, MA, USA) according to the manufacturer’s protocol.

Purified DNA from all cell types was analyzed by quantitative PCR using THUNDERBIRD Next SYBR qPCR Mix on a LightCycler 96 system (Roche Diagnostics, Basel, Switzerland). Primers specific for the promoter regions of target genes were designed. The primer sequences for the ERBB3 promoter were as follows:

Forward: 5′-GTGGCTCTTGCCTCGATGT-3′

Reverse: 5′-GCAGAGGGTGAAGGGAGC-3′

Each qPCR reaction was performed in triplicate. Data were analyzed using the ΔΔCt method. Enrichment of target sequences was normalized to input DNA and presented as fold enrichment over the IgG negative control. Statistical analysis was performed using GraphPad Prism 8.0 software. Data are presented as mean ± standard deviation from at least three independent experiments. Statistical significance was determined using a two-tailed unpaired *t*-test, with *p* < 0.05 considered statistically significant.

### 2.14. Protein Extraction and Western Blot Analysis

Total protein was extracted from cells after washing three times with PBS. Cells were lysed in ice-cold lysis buffer (50 mM Tris-HCl pH 7.4, 150 mM NaCl, 1% NP-40, 0.1% SDS) supplemented with PhosSTOP phosphatase inhibitor cocktail and cOmplete™ Mini protease inhibitor cocktail tablets (Roche Diagnostics, Basel, Switzerland). Whole cell lysates were centrifuged at 12,000× *g* at 4 °C, and the supernatant was retained. Protein concentration was determined using the BCA Protein Assay Kit and adjusted to 2 μg/μL with lysis buffer and 4X SDS sample buffer. Proteins were separated by SDS-PAGE and transferred to PVDF membranes (LI-COR Biosciences, Lincoln, NE, USA) following standard procedures. Membranes were blocked for 1 h at room temperature in Odyssey Blocking Buffer (LI-COR Biosciences, Lincoln, NE, USA). Primary antibodies were diluted in Tris-Buffered Saline with 0.1% Tween 20 (TBST) containing 0.1% BSA (Sigma Aldrich, St. Louis, MO, USA) and incubated with the membranes overnight at 4 °C. After washing three times with TBST (5 min each), membranes were incubated with IRDye^®^ 680RD Goat anti-Rabbit IgG and IRDye^®^ 800CW Goat anti-Mouse IgG secondary antibodies (LI-COR Biosciences, Lincoln, NE, USA) diluted in blocking buffer for 1 h at room temperature. Following three additional 5-min washes with TBST, protein bands were visualized using the ODYSSEY Fc imaging system (LI-COR Biosciences, Lincoln, NE, USA).

### 2.15. Statistical Analysis

All statistical tests were performed using GraphPad Prism 9 (GraphPad Prism 9.5.1). The statistical analysis details for each experiment can be found in figure legends and in the method details section.

## 3. Results

### 3.1. ARID2 KO Enhances Tumor Progression in TFE3-Rearranged Renal Cell Carcinoma

**Figure 1:**
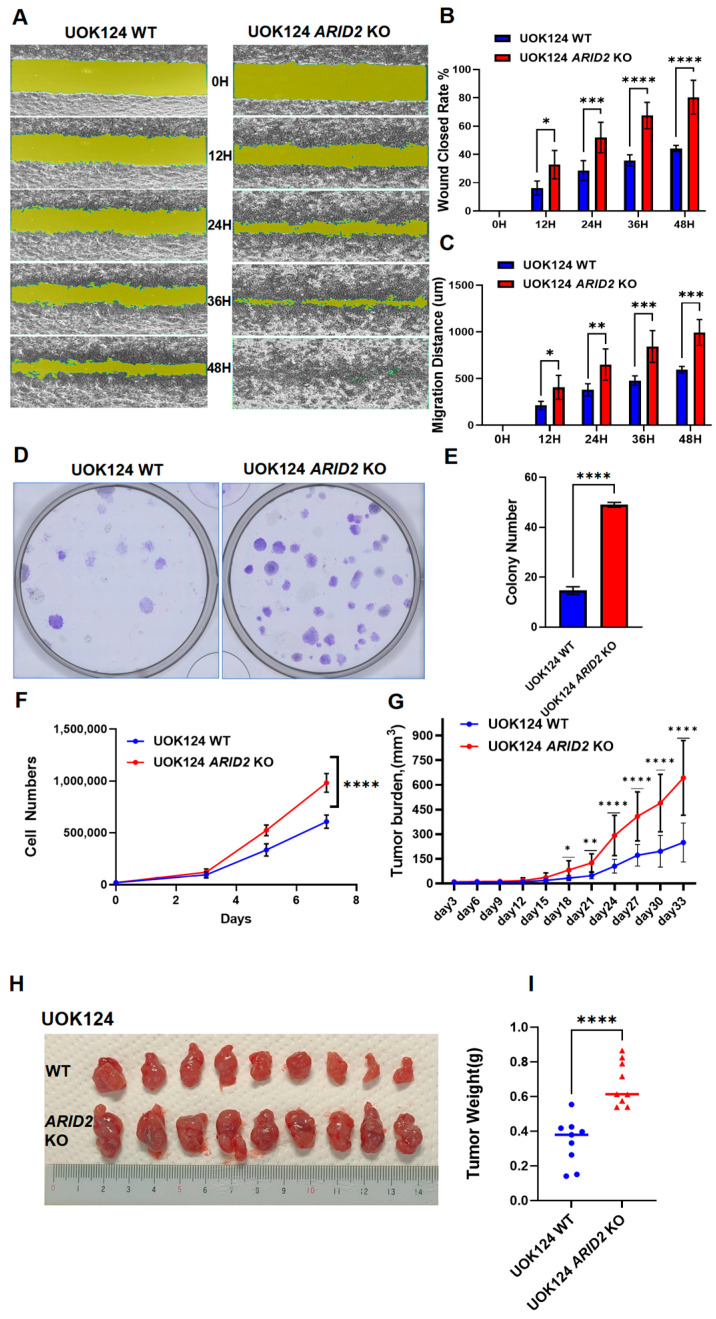
*ARID2* KO enhances tumor progression in *TFE3*-rearranged Renal Cell Carcinoma. (**A**) The effect of *ARID2* KO on the migration of TFE3-RCC UOK124 cells. Determined by cell scratch assay are set at five time points: 0 h, 12 h, 24 h, 36 h, and 48 h. (**B**) Wound closure rate. (**C**) Migration distance. Data were presented as mean ± SEM of three individual experiments. *p* values were determined by multiple unpaired *t*-test. (**D**,**E**) Effect of *ARID2* KO on colony formation of UOK124 cells. At least three biological replicates were performed for each condition. (**F**) Effect of *ARID2* KO on the proliferation of TFE3-RCC UOK124 cells. Data were presented as mean ± SEM of three individual experiments. *p* values were determined by unpaired *t*-test. (**G**–**I**) The UOK124 and UOK124 *ARID2* KO cells were injected into the subcutaneous tissue of nude mice. Starting on the third day after injection, tumor volume was measured every three days. Tumor volumes were measured and plotted as mean ± SEM (*n* = 5 animals per group). *p* values were determined by unpaired *t*-test (**G**). On the 33rd day, when the diameter of one of the tumors grew to nearly but not exceeding 20 mm, the tumors were harvested by sacrificing the mice. (**H**) Tumors were weighted and plotted as mean ± SEM (n = 5 animals per group). *p* values were determined by unpaired *t*-test. (**I**) *p* > 0.05; * *p* < 0.05; ** *p* < 0.01; *** *p* < 0.001; **** *p* < 0.0001.

To thoroughly investigate the role of *ARID2* in *TFE3*-rearranged renal cell carcinoma (TFE3-RCC) cells, we designed a series of in vitro and in vivo experiments. We selected the UOK124 cell line, which was established from human TFE3-RCC and contains the *PRCC-TFE3* fusion gene. This cell line expresses ARID2 and exhibits aggressive behavior, allowing us to investigate how *ARID2* loss might further enhance aggressiveness in *TFE3*-rearranged RCC. First, the *ARID2* gene was knocked out in the UOK124 cell line, which was established from human TFE3-RCC and contains the *PRCC-TFE3* fusion gene (Supplementary Figure S1). The effects of the *ARID2* KO were then assessed using cell migration assays. The *ARID2* KO group not only exhibited faster wound healing but also demonstrated significantly increased migration distances compared to the UOK124 *ARID2* WT group (Figure 1A–C). This finding strongly suggests that *ARID2* may play a crucial role in suppressing the migration of TFE3-RCC cells. Furthermore, we conducted cell colony formation and proliferation assays. The results revealed that the UOK124 *ARID2* KO group not only formed significantly more colonies (Figure 1D, E) but also demonstrated a higher cell proliferation rate compared to the UOK124 WT group (Figure 1F). These in vitro experimental results consistently point to *ARID2*’s tumor-suppressive function. To verify whether this finding holds true in an in vivo environment, we injected UOK124 *ARID2* KO cells and UOK124 *ARID2* WT cells into nude mice. As the experiment progressed, the differences between the two groups became increasingly apparent, reaching statistical significance by day 18 (Figure 1G,H). Furthermore, the dissection results at the conclusion of the experiment (day 33) revealed that tumors in the UOK124 *ARID2* KO group were significantly heavier than those in the UOK124 *ARID2* WT group (Figure 1I). In conclusion, these multifaceted and mutually corroborating experimental results strongly support our hypothesis: *ARID2* likely plays a key tumor-suppressive role in TFE3-RCC.

### 3.2. ERBB3 Emerges as a Key Common Target for PRCC-TFE3 Chimeric Protein and ARID2

**Figure 2:**
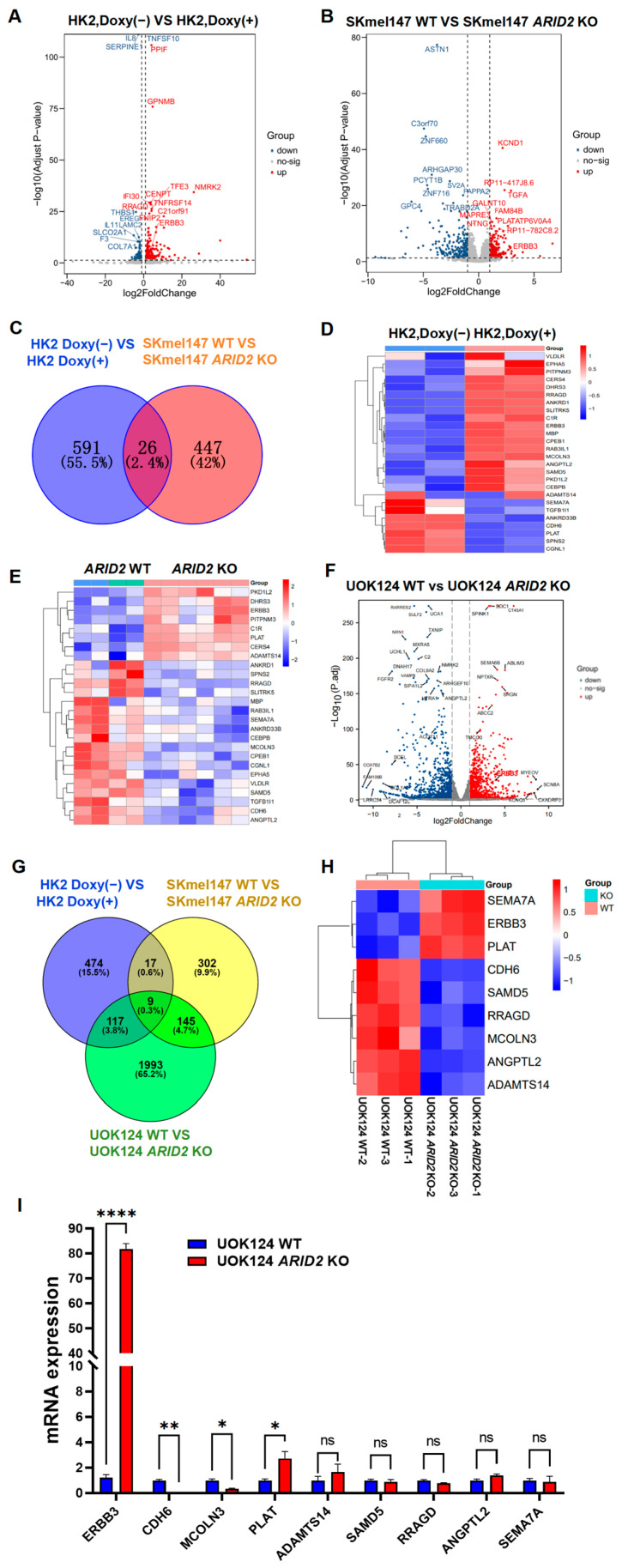
ERBB3 emerges as a key common target for PRCC-TFE3 chimeric protein and *ARID2*. (**A**) The volcano plot illustrates the distribution and expression of DEGs between PRCC-TFE3 doxycycline-inducible HK2 cells cultured without doxycycline and with doxycycline. The data processing was based on the criteria of log2(fold change) > 1 and *p*.adj-value < 0.05. (**B**) Volcano plot is derived from the differences between SKmel147 WT cells vs. SKmel147 *ARID2* KO cells. The data processing was based on the criteria of log2(fold change) > 1 and *p*.adj-value < 0.05 (**B**). (**C**) A Venn diagram was used to analyze the intersection of two sets of DEGs (PRCC-TFE3 inducible HK2 without doxycycline vs. with doxycycline and SKmel147 WT vs. SKmel147 *ARID2* KO), revealing 26 genes that are commonly regulated in PRCC-TFE3 expressing HK2 and SKmel147 *ARID2* KO. (**D**) Heatmap of the commonly regulated 26 genes from the Venn data (**C**), based on PRCC-TFE3 non-induced HK2 vs. PRCC-TFE3 induced HK2. (**E**) Heatmap of the commonly regulated 26 genes from the Venn data (**C**), based on SKmel147 WT and SKmel147 *ARID2* KO cells. (**F**) The volcano plot illustrates the distribution and expression of DEGs. The data are derived from the differences between UOK124 WT cells and UOK124 *ARID2* KO cells. The data processing was based on the criteria of log2(fold change) > 1 and *p*.adj-value < 0.05. (**G**) The DEGs from the three groups: HK2 Doxy(−) vs. HK2 Doxy(+), SKmel147 WT vs. SKmel147 *ARID2* KO, and UOK124 WT vs. UOK124 *ARID2* KO, were analyzed using a Venn diagram. A total of nine DEGs were found to be commonly regulated among the three groups. (**H)** Heatmap of the commonly regulated nine genes from Venn data (**G**), based on UOK124 WT vs. UOK124 *ARID2* KO. (**I**) RNA was extracted from UOK124 WT cells and UOK124 *ARID2* KO cells, and qPCR analysis was performed on nine commonly regulated genes, revealing significant differences in ERBB3 expression. Ct values were analyzed using the 2^−ΔΔCt^ method. Data were presented as mean ± SEM of three individual experiments, *p* values were determined by two tail unpaired *t*-test. ns, *p* > 0.05; * *p* < 0.05; ** *p* < 0.01; **** *p* < 0.0001.

To thoroughly investigate the molecular mechanism by which *ARID2* loss contributes to TFE3-RCC progression, we designed a series of experiments and conducted comprehensive gene expression analyses. First, we used a doxycycline (doxy) induction system to express the PRCC-TFE3 fusion gene in HK-2 human renal proximal tubule cells. RNA sequencing analysis identified 591 differentially expressed genes (DEGs) in PRCC-TFE3 non-induced (Doxy(−)) and induced (Doxy(+)) conditions (Figure 2A). This finding provides important clues for describing the target genes of the PRCC-TFE3 chimeric transcription factor. Next, to explore the impact of *ARID2* KO, we referenced the study by Saul Carcamo et al. [23], which identified 447 DEGs in SKmel147 WT and SKmel147 *ARID2* KO melanoma cells (Figure 2B). This comparison provides valuable insights into the transcriptional changes induced by *ARID2* loss, which may be of relevance to our investigation of *ARID2*’s role in TFE3-RCC. To identify genes commonly regulated by PRCC-TFE3 and *ARID2*, we performed a Venn diagram analysis, which revealed 26 shared genes (Figure 2C–E). Among these 26 genes, 6 were commonly up-regulated in both PRCC-TFE3-induced HK-2 cells and *ARID2* KO SKmel147 cells (Figure 2D,E). Of these six genes, the *ERBB3* proto-oncogene emerged as a particularly noteworthy candidate potentially responsible for the oncogenic progression driven by *ARID2* KO in TFE3-RCC. To further validate this hypothesis, we performed RNA sequencing on UOK124 WT and UOK124 *ARID2* KO cells. The results revealed that *ARID2* KO led to 2264 differentially expressed genes, with *ERBB3* once again identified as a significantly up-regulated gene (Figure 2F). More significantly, our comprehensive analysis of DEGs across the three cell line groups identified nine shared genes, with *ERBB3* among them. (Figure 2G). Finally, heatmap analysis and qPCR validation further confirmed the consistent up-regulation of ERBB3 in *ARID2*-deficient TFE3-RCC cells. (Figure 2H,I). Our study reveals the extensive impact of the PRCC-TFE3 fusion gene and *ARID2* knockout on cellular gene expression. Additionally, it crucially identifies ERBB3 as a common downstream target of these genetic alterations.

### 3.3. PRCC-TFE3 Fusion Protein Expression and ARID2 Loss Synergistically Up-Regulate ERBB3 Expression

**Figure 3:**
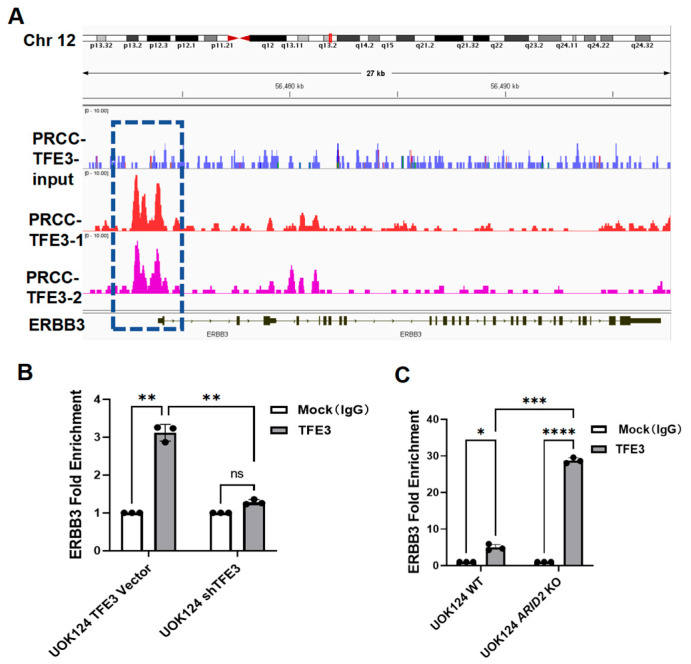
PRCC-TFE3 fusion protein expression and *ARID2* loss synergistically up-regulate ERBB3 expression. (**A**) Results from two biological replicates of HA-TFE3 ChIP-seq in PRCC-TFE3 doxycycline-inducible HK2 cells. PRCC-TFE3 binding is seen along the promoter regions of *ERBB3* gene. Dashed box marks *ERBB3* binding peaks. (**B**) ChIP-qPCR analysis demonstrates the binding of endogenous PRCC-TFE3 to the promoter region of *ERBB3* in UOK124 vector cells, which was significantly abolished in PRCC-TFE3-knockdown UOK124 cells. (**C**) ChIP-qPCR analysis demonstrates the binding of endogenous PRCC-TFE3 to the promoter region of *ERBB3* in UOK124 cells, which was significantly increased in *ARID2* KO UOK124 cells. *p* values were determined by two-way ANOVA test. ns, *p* > 0.05; * *p* < 0.05; ** *p* < 0.01; *** *p* < 0.001; **** *p* < 0.0001.

Next, we considered the possibility that *ERBB3* is a direct transcriptional target of PRCC-TFE3. To investigate this, we analyzed our chromatin immunoprecipitation sequencing (ChIP-seq) data obtained from HK-2 cells expressing PRCC-TFE3. Distinct PRCC-TFE3 binding peaks were observed in the promoter region of *ERBB3*, suggesting that PRCC-TFE3 directly regulates ERBB3 expression (Figure 3A). To further validate PRCC-TFE3 binding to the *ERBB3* gene at the endogenous protein level, we performed chromatin immunoprecipitation followed by quantitative PCR (ChIP-qPCR) on UOK124 cells and their TFE3-knockdown counterparts. The results confirmed the binding of endogenous PRCC-TFE3 to the *ERBB3* gene (Figure 3B). Integrating these findings with the transcriptome data of HK-2 cells (Figure 2A), it is strongly suggested that the PRCC-TFE3 fusion protein directly binds to and up-regulates the expression of the proto-oncogene *ERBB3* in TFE3-RCC. Furthermore, the up-regulation of ERBB3 expression observed in *ARID2* KO cells (Figure 2B,F,I) suggests that *ARID2* likely plays a role in regulating ERBB3 expression as well. The loss of *ARID2* results in the complete dissolution of the PBAF chromatin-remodeling complex, potentially leading to alterations in chromatin accessibility around the *ERBB3* gene. These changes may enhance the accessibility of the *ERBB3* gene to PRCC-TFE3. To investigate this hypothesis, we compared endogenous PRCC-TFE3 binding to the *ERBB3* gene using ChIP-qPCR in UOK124 WT cells and UOK124 *ARID2* KO cells. As shown in Figure 3B, PRCC-TFE3 exhibits significant binding to the *ERBB3* promoter in UOK124 WT cells. Notably, in UOK124 *ARID2* KO cells, this binding is dramatically enhanced compared to UOK124 WT cells (Figure 3C). The loss of *ARID2* significantly amplifies PRCC-TFE3’s affinity for the *ERBB3* promoter, resulting in a marked increase in PRCC-TFE3 occupancy at this genomic locus. This enhanced binding suggests that *ARID2* deficiency synergistically potentiates PRCC-TFE3-mediated ERBB3 expression by facilitating PRCC-TFE3 interactions with the *ERBB3* gene regulatory region.

### 3.4. ERBB3 Signaling Pathway Enrichment in ARID2-Deficient TFE3-Rearranged RCC

**Figure 4:**
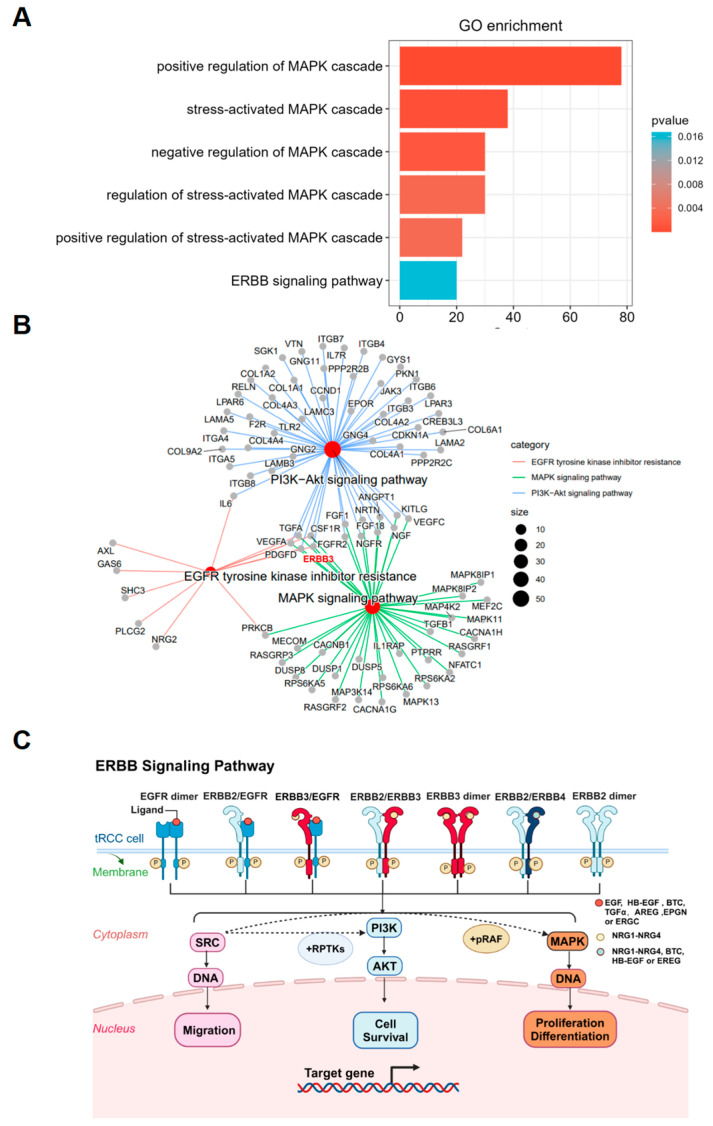
ERBB3 signaling pathway enrichment in *ARID2*-deficient *TFE3*-rearranged RCC. (**A**) GO functional enrichment analysis of DEGs in UOK124 WT cells and UOK124 *ARID2* KO cells. (**B**) KEGG net pathway enrichment analysis of DEGs in UOK124 WT cells and UOK124 *ARID2* KO cells. (**C**) Schematic illustration of ERBB signaling pathway.

To better understand how *ARID2* deficiency impacts these signaling pathways, RNA-seq data from UOK124 WT and UOK124 *ARID2* KO cells were subjected to further bioinformatic analysis. Gene ontology (GO) analysis of DEGs between UOK124 WT and UOK124 *ARID2* KO cells revealed significant enrichment in MAPK and ERBB3 signaling pathways, strongly indicating the pivotal role of these pathways in the context of *ARID2* deficiency (Figure 4A). Furthermore, KEGG network analysis demonstrated that the *ERBB3* gene is significantly enriched together in the gene sets of the PI3K-Akt and MAPK signaling pathways, as well as in the pathways related to EGFR tyrosine kinase inhibitor resistance. (Figure 4B).

ERBB3, a key member of the EGFR family, primarily functions by forming heterodimers with other EGFR family proteins. EGFR enhances signal transduction activity by forming heterodimers with ERBB3, and its activity level is higher than that of the ERBB3 homodimer [42,43,44]. Upon phosphorylation, ERBB3 provides optimal binding sites for SRC family kinases (SFKs), initiating a complex positive feedback loop that significantly amplifies signal strength, rather than merely facilitating a simple protein interaction. This signal amplification mechanism triggers two parallel yet interconnected signaling pathways. First, SRC kinases activate PI3K, leading to AKT activation, which plays a crucial role in regulating cell proliferation and survival. Second, ERBB3 and SRC synergistically activate RAS, initiating the RAF-MEK-ERK cascade. The coordinated action of these two pathways profoundly influences gene transcription and cellular phenotype, exemplifying the intricacy and precision of cellular signaling networks (Figure 4C). In subsequent investigations, we will explore the specific effects of ERBB3 in the context of *ARID2* KO in TFE3-RCC and elucidate its regulatory mechanisms on key downstream target genes within these signaling pathways.

### 3.5. Selective ERBB3 Up-Regulation and Downstream Pathway Activation in ARID2-Deficient TFE3-RCC Cells

**Figure 5:**
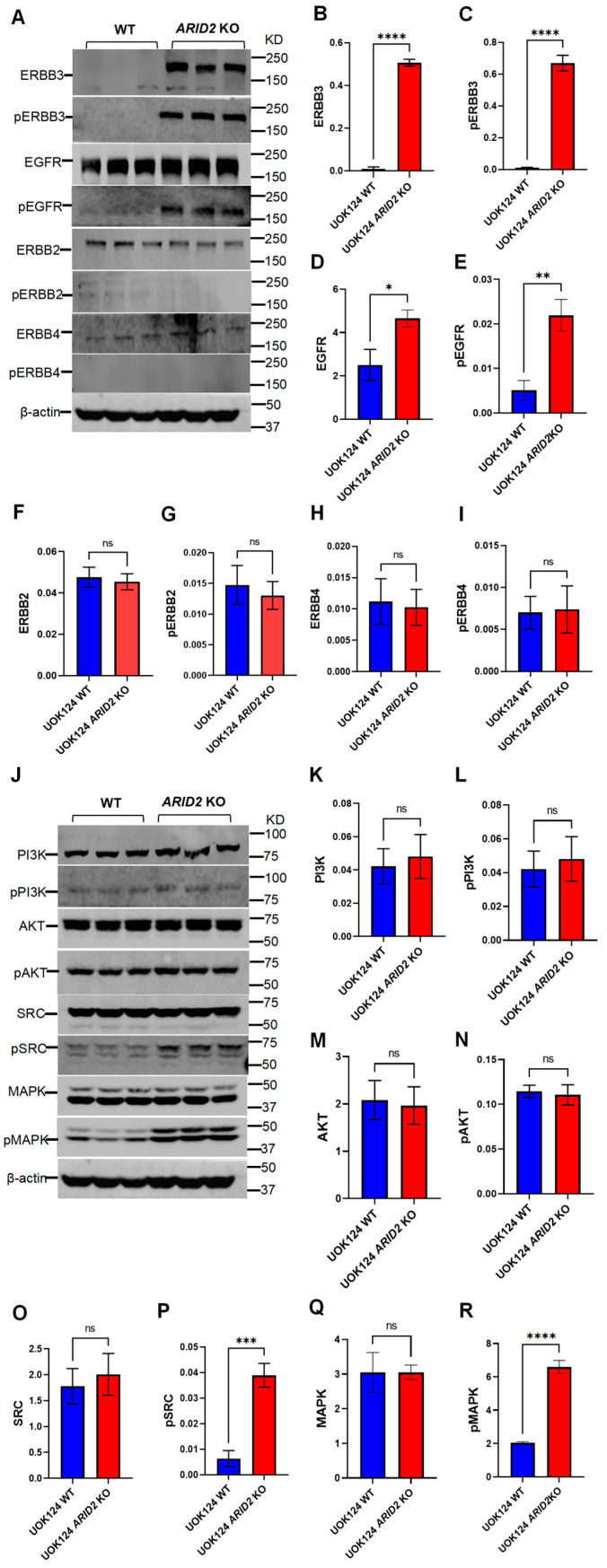
Selective ERBB3 Up-regulation and Downstream Pathway Activation in *ARID2*-Deficient TFE3-RCC Cells. (**A**) Immunoblot of ERBB3, pERBB3, EGFR, pEGFR, ERBB2, pERBB2, ERBB4, pERBB4 in UOK124 WT and UOK124 *ARID2* KO cells. β-actin was used as a loading control, (n = 3). (**B**–**I**) The protein expression levels of ERBB3, pERBB3, EGFR, pEGFR, ERBB2, pERBB2, ERBB4, and pERBB4 were quantified using Image Studio 6.0 software on Western blotting images obtained by Odyssey XF. Data were presented as mean ± SEM of three individual experiments, *p* values were determined by two-tail unpaired *t*-test. ns, *p* > 0.05; * *p* < 0.05; ** *p* < 0.01; *** *p* < 0.001; **** *p* < 0.0001. (**J**) Immunoblot of PI3K, pPI3K, AKT, pAKT, SRC, pSRC, MAPK, pMAPK in UOK124 WT, and UOK124 *ARID2* KO cells. β-actin was used as a loading control, (n = 3). (**K**–**R**) The protein expression levels of PI3K, pPI3K, AKT, pAKT, SRC, pSRC, MAPK, pMAPK were quantified using Image Studio 6.0 software on Western blotting images obtained by Odyssey XF. Data were presented as mean ± SEM of three individual experiments, *p* values were determined by two-tail unpaired *t*-test. ns, *p* > 0.05; * *p* < 0.05; ** *p* < 0.01; *** *p* < 0.001; **** *p* < 0.0001.

As the transcriptome data showed significant differences in *ERBB3* gene expression, this prompted us to further explore changes at the protein level. Through systematic protein analysis, we found that both total ERBB3 and phosphorylated ERBB3 were significantly up-regulated in UOK124 *ARID2* KO cells (Figure 5A–C). This result suggests that ERBB3 may play a crucial role in the phenotypic alterations observed in UOK124 cells as a consequence of *ARID2* deficiency. To comprehensively understand the role of the ERBB family in this process, we simultaneously examined the expression of other ERBB members. Interestingly, while EGFR exhibited elevated protein and phosphorylation levels in UOK124 *ARID2* KO cells (Figure 5A,D,E), no significant differences were observed in ERBB2/HER2 (Figure 5A,F,G) or ERBB4/HER4 (Figure 5A,H,I) levels. This selective alteration in expression further underscores the unique status of ERBB3 in the context of *ARID2* deficiency in TFE3-RCC. Subsequently, we shifted our research focus to the downstream signaling pathways of ERBB3. Despite the up-regulation of ERBB3 expression, we observed no significant changes in the activity of PI3K (Figure 5J–L) or AKT (Figure 5J,M,N). In contrast, we detected a significant increase in the levels of phosphorylated SRC (pSRC; Figure 5J,O,P) and phosphorylated MAPK (pMAPK; Figure 5J,Q,R) in UOK124 *ARID2* KO cells.

### 3.6. Specific Effects of ERBB3 Inhibitors in the Context of UOK124 ARID2 Deficiency

**Figure 6:**
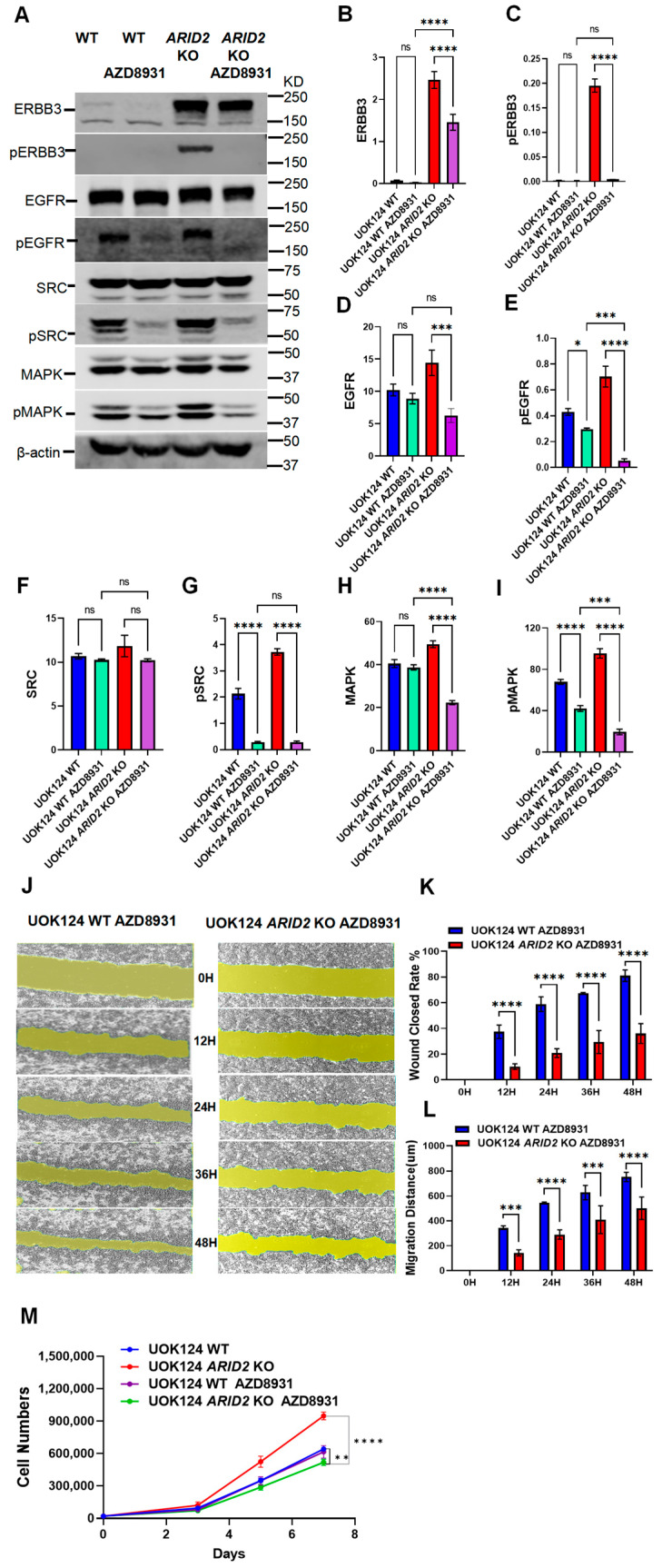
Specific Effects of ERBB3 Inhibitors in the Context of UOK124 *ARID2* Deficiency. (**A**) Immunoblot of ERBB3, pERBB3, EGFR, pEGFR, SRC, pSRC, MAPK, pMAPK, in UOK124 WT and UOK124 *ARID2* KO cells cultured with and without ERBB3 inhibitor (AZD8931, Cat. No. S2192). β-actin was used as a loading control, (n = 3). (**B**–**I**) The protein expression levels of ERBB3, pERBB3, EGFR, pEGFR, SRC, pSRC, MAPK, pMAPK were quantified using Image Studio 6.0 software on Western blotting images obtained by Odyssey XF. Data were presented as mean ± SEM of three individual experiments, *p* values were determined by two-tail unpaired *t*-test. ns, *p* > 0.05; * *p* < 0.05; *** *p* < 0.001; **** *p* < 0.0001. (**J**) The effect of an ERBB3 inhibitor on the migration of UOK124 WT and UOK124 *ARID2* KO cells. Determined by cell scratch assays set at five time points: 0 h, 12 h, 24 h, 36 h, and 48 h. (**K**) Wound closure rate. (**L**) Migration distance. Data were presented as mean ± SEM of three individual experiments. *p* values were determined by multiple unpaired *t*-test, *p* > 0.05 is significant. *** *p* < 0.001; **** *p* < 0.0001. (**M**) Cell proliferation curves of UOK124 WT and UOK124 *ARID2* KO cells cultured with or without an ERBB3 inhibitor. Data were presented as mean ± SEM of three individual experiments. *p* values were determined by unpaired *t*-test. ** *p* < 0.01; **** *p* < 0.0001.

To explore the therapeutic potential of ERBB3 inhibitor (AZD8931) in *ARID2*-deficient TFE3-RCC, we evaluated the effects of the AZD8931 through Western blot analysis. Our results demonstrate that while UOK124 WT cells showed no significant changes, *ARID2* KO cells exhibited significant decreases in ERBB3, EGFR, and MAPK protein levels (with the exception of SRC) upon AZD8931 treatment (Figure 6A,B,D,F,H). Notably, the phosphorylated forms of ERBB3 and EGFR, along with their downstream targets pSRC and pMAPK, exhibited a more pronounced decrease in *ARID2* KO cells (Figure 6A,C,E,G,I). These findings strongly suggest that *ARID2*-deficient cells demonstrate greater sensitivity to AZD8931 compared to WT cells.

To determine whether these molecular-level changes translate into functional differences, we conducted scratch wound healing assays. The results demonstrated that UOK124 *ARID2* KO cells exhibited significant functional reversal following AZD8931 treatment (Figure 6J). This reversal was specifically manifested as lower wound closure rates (Figure 6K) and shorter migration distances (Figure 6L) compared to UOK124 WT cells. This finding strongly suggests that UOK124 *ARID2* KO cells heavily rely on ERBB3 for their migratory capabilities.

Finally, cell proliferation studies provided decisive evidence for our conclusions. Compared to UOK124 wild type, the UOK124 *ARID2* KO group proliferated faster (**** *p* < 0.0001). However, when treated with the inhibitor (AZD8931), the *ARID2* KO group’s proliferation rate significantly slowed down (** *p* < 0.01), becoming the slowest among all experimental groups (Figure 6M). This result underscores the critical role of ERBB3 in the context of *ARID2*-deficient TFE3-RCC.

## 4. Discussion

Our study explored the functional consequence of *ARID2* deficiency in TFE3-RCC through a series of in vitro and in vivo experiments. First, we confirmed the tumor-suppressive role of *ARID2* in TFE3-RCC. *ARID2* KO led to enhanced migration, increased colony formation, and accelerated proliferation of TFE3-RCC cells. In vivo experiments further validated that *ARID2* KO promoted tumor growth. These findings strongly support the hypothesis that the loss of *ARID2* function may synergize with the oncogenic fusion TFE3, contributing to the progression of TFE3-RCC. Comprehensive transcriptomic analysis revealed ERBB3 as a key target regulated by both the *PRCC-TFE3* fusion gene and *ARID2* KO. ChIP-seq and ChIP-qPCR experiments confirmed direct binding of PRCC-TFE3 to the *ERBB3* regulatory region. Notably, *ARID2* KO significantly increased PRCC-TFE3 binding to this region, indicating a synergistic regulatory mechanism between PRCC-TFE3 and *ARID2* deficiency. These findings indicate that chromatin remodeling resulting from *ARID2* loss likely alters DNA accessibility for PRCC-TFE3, leading to ERBB3 over-expression. Indeed, Carcamo et al. demonstrated that *ARID2* depletion in melanoma cells leads to a complete loss of the PBAF complex, which may result in the redistribution of the remaining SWI/SNF core subunits to the BAF complex. This redistribution allows the BAF complex to relocate to regions of open chromatin, enhancing chromatin accessibility and facilitating transcription factor binding, including MITF [23]. We then investigated the changes in the ERBB3 signaling pathway in the context of *ARID2* deficiency. RNA-seq analysis revealed significant enrichment of differentially expressed genes in the MAPK and ERBB3 signaling pathways when comparing *ARID2*-wild type and *ARID2*-KO TFE3-RCC cells. Western blot analysis revealed that *ARID2* KO led to the up-regulation and activation of both ERBB3 and EGFR, a finding of particular significance. While ERBB3 itself possesses low intrinsic kinase activity, its signaling capacity is dramatically enhanced through heterodimerization with EGFR [42,43,44]. The observed up-regulation of both total EGFR and its phosphorylated form (pEGFR) in ERBB3-expressing, *ARID2*-KO TFE3-RCC cells strongly suggests the formation of ERBB3-EGFR heterodimers. This heterodimerization likely amplifies downstream signaling pathways, potentially contributing to the aggressive phenotype observed in *ARID2*-deficient TFE3-RCC cells. Regarding downstream signaling, selective activation of the MAPK and SRC pathways, rather than the canonical PI3K/AKT pathway, was observed in *ARID2*-KO TFE3-RCC cells. This unexpected pattern of pathway activation suggests a unique signaling profile in these cells, which may have important implications for understanding the disease mechanism and developing targeted therapies for *ARID2*-deficient TFE3-RCC.

Finally, we investigated the potential therapeutic efficacy of the ERBB3 inhibitor AZD8931 in *ARID2* KO TFE3-RCC cells. Our experimental results revealed that *ARID2* KO cells exhibited greater sensitivity to AZD8931 compared to *ARID2* WT cells, demonstrated by significant reductions in protein levels associated with ERBB3 signaling, including phospho-EGFR and phospho-MAPK, along with a marked inhibition of cell migration and proliferation. These findings indicate that *ARID2* KO TFE3-RCC cells are highly dependent on ERBB3 signaling, providing crucial experimental support for the potential use of targeted therapy in *ARID2*-deficient TFE3-RCC patients. In conclusion, this study reveals the mechanism by which *ARID2* deficiency promotes TFE3-RCC progression through up-regulation of ERBB3 expression and selective activation of downstream signaling pathways. These findings not only deepen our understanding of the molecular mechanisms of TFE3-RCC but also provide new avenues for precision treatment of *ARID2*-deficient TFE3-RCC. Future research should focus on elucidating the molecular mechanisms by which fusion TFE3 and *ARID2* loss synergistically contribute to the over-expression of ERBB3, and on exploring the clinical potential of these findings.

While our study provides valuable insights into *ARID2*’s role in TFE3-RCC, we acknowledge limitations, particularly in the context of using the UOK124 cell line. Although ARID2 is expressed in UOK124, which is a cell line with high malignancy, our experiment involved knocking out *ARID2* in this cell line. For a more convincing evaluation of *ARID2*’s function as a tumor suppressor gene in the future, restoring *ARID2* in *ARID2*-deficient TFE3-RCC cell lines would be more persuasive. Future studies should employ a broader panel of cell lines, including both *ARID2*-deficient and *ARID2*-positive TFE3-RCC cells, to offer a more comprehensive view of *ARID2*’s function across different stages of TFE3-RCC progression.

*ARID2* mutations are observed in approximately 4.55% of TFE3-RCC cases [16]. Given this relatively low mutation frequency, we have acknowledged the limitations of our current study and outlined future research directions to further explore ARID2 expression in TFE3-RCC clinical samples. Although the sample size of TFE3-RCC (n = 15) in The Cancer Genome Atlas (TCGA) is small, there is a subtle trend indicating that lower ARID2 expression may be associated with poorer prognosis (Supplemental Figure S2). Conducting analyses with a larger cohort of TFE3-RCC samples remains an important task for future studies to draw statistically significant conclusions. Furthermore, assessing ARID2 expression at the protein level through immunohistochemistry in TFE3-RCC clinical samples would be a valuable future study to evaluate the significance of this research.

## 5. Conclusions

*ARID2* plays a crucial tumor-suppressive role in *TFE3*-rearranged Renal Cell Carcinoma (TFE3-RCC). Our research highlights ERBB3 as a key target influenced by both the PRCC-TFE3 fusion protein and *ARID2* loss. The up-regulation of ERBB3 in *ARID2*-deficient cells indicates that the absence of *ARID2* promotes tumor progression through a critical pathway, underscoring its importance in maintaining cellular homeostasis and preventing tumorigenesis. Furthermore, our study demonstrates that the loss of *ARID2*, which is known to disrupt the PBAF chromatin remodeling complex, enhances PRCC-TFE3 binding to the *ERBB3* promoter, thereby increasing ERBB3 expression mediated by PRCC-TFE3. This synergistic effect leads to significant activation of downstream signaling pathways such as MAPK and SRC, further promoting tumor growth and migration. The therapeutic potential of ERBB3 inhibitors, such as AZD8931, demonstrates that TFE3-RCC cells lacking *ARID2* are more sensitive to this treatment, providing promising directions for improving outcomes for patients with this aggressive cancer subtype. These findings not only reveal the critical role of *ARID2* in TFE3-RCC but also offer a potential basis for developing new targeted therapies.

## Figures and Tables

**Table 1:** The antibodies used in this study.

Antibodies and Source	Cat. No.
ARID2 (D8D8U) Rabbit mAb	#82324
EGF Receptor (D38B1) XP^®^ Rabbit mAb	#4267
(Tyr1284)/EGFR (Tyr1173) (21A9) Rabbit mAb	#4757
Receptor (Tyr1068)(D7A5)XP^®^ Rabbit mAb Phospho EGF	#8543
HER2/ErbB2 (D8F12) XP^®^ Rabbit mAb	#4290
HER3/ErbB3 (D22C5) XP^®^ Rabbit mAb	#12708
HER4/ErbB4 (111B2) Rabbit mAb	#4795
Phospho-HER2/ErbB2 (Tyr1221/1222) Rabbit mAb	#2243
Phospho-HER3/ErbB3 (Tyr1289) (D1B5) Rabbit mAb	#2842
Phospho-HER4/ErbB4 Akt Antibody	#9272
Src (36D10) Rabbit mAb	#2109
Phospho-Src Family (Tyr416) Antibody	#2101
PI3 Kinase p85 (19H8) Rabbit mAb	#4257
Phospho-PI3 Kinase p85/p55 Rabbit mAb	#17366
p44/42 MAPK (Erk1/2) (137F5) Rabbit mAb	#4695
Phospho-p44/42 MAPK (Erk1/2) XP^®^ Rabbit mAb	#4370
Beta Actin Polyclonal antibody Rabbit IgG	#20536

All antibodies used in this study were sourced from Cell Signaling Technology (Danvers, MA, USA), with the exception of the Beta Actin Polyclonal antibody. This particular antibody was obtained from Proteintech (San Diego, CA, USA).

**Table 2:** Gene primer information.

Primer Probe Name	Sequence
ERBB3 Forward	5′-CTATGAGGCGATACTTGGAACGG-3′
ERBB3 Reverse	5′-GCACAGTTCCAAAGACACCCGA-3′
ADAMTS14 Forward	5′-TGAAGGCGGATGACAAGTGTGG-3′
ADAMTS14 Reverse	5′-CAGTGCCTCAATCTGGATGTGC-3′
CDH6 Forward	5′-AGATGCTGCCAGGAATCCTGTC-3′
CDH6 Reverse	5′-CCATAGCAGTGTTTCTCGGTCAA-3′
SEMA7A Forward	5′-CTTCTTCCGAGAGGACAATCCTG-3′
SEMA7A Reverse	5′-GTGTTCCACTTGGAGACTGACAG-3′
RRAGD Forward	5′-CGATGACCTTGCAGATGCTGGA-3′
RRAGD Reverse	5′-AGATGTTCAGCAAATTCTCCAGAG-3′
ANGPTL2 Forward	5′-AGACGCCTGGATGGCTCTGTTA-3′
ANGPTL2 Reverse	5′-AGTTGCCTTGGTTCGTCAGCCA-3′
SAMD5 Forward	5′-TCCTTCGTGGATAACGGCTACG-3′
SAMD5 Reverse	5′-GTTGGCGTCCTGCTCCCGCA-3′
MCOLN3 Forward	5′-ATGCTCGTGTGGCTTGGAGTCA-3′
MCOLN3 Reverse	5′-CCATCCACAGAAGCAGTAACCTA-3′
PLAT Forward	5′-TGGTGCTACGTCTTTAAGGCGG-3′
PLAT Reverse	5′-GCTGACCCATTCCCAAAGTAGC-3′

## Data Availability

The original data presented in this study are publicly available in Gene Expression Omnibus with the accession numbers GSE278984, GSE279136, and GSE281281. RNA sequencing data of *ARID2* KO versus WT SKmel147 cells (parent), presented in the study are openly available in GSE172383.

## Supplementary Materials

The following supporting information can be downloaded at: https://www.mdpi.com/article/10.3390/cimb46120817/s1, Figure S1: Validation of ARID2knockout in UOK124 cell lines by western blot analysis.; Figure S2: Kaplan-Meier analysis of overall survival based on ARID2 mRNA expression levels in TFE3-RCC patients from TCGA cohorts.

## Abbreviations

TFE3-RCC	*TFE3*-rearranged Renal Cell Carcinoma
MITF	Microphthalmia-associated transcription factor
ChIP	Chromatin immunoprecipitation
BAF	BRG1/BRM-associated factor
PBAF	Polybromo-associated BAF
ccRCC	Clear cell Renal Cell Carcinoma
LUAD	Lung adenocarcinoma
BLCA	Bladder urothelial carcinoma
SKCM	Skin cutaneous melanoma
NSCLC	Non-small cell lung cancer
HCC	Hepatocellular carcinoma
