# Supplementary material for: ARID2 Deficiency Enhances Tumor Progression via ERBB3 Signaling in TFE3-Rearranged Renal Cell Carcinoma
Source: Curr Issues Mol Biol. 2024 Dec 2;46(12):13675–95. doi: 10.3390/cimb46120817 (PMC11727593; doi:10.3390/cimb46120817)
Supplement: Supplementary file 1 [file cimb-46-00817-s001.zip › cimb-3303861-supplementary.pdf]

Supplemental Figure S1

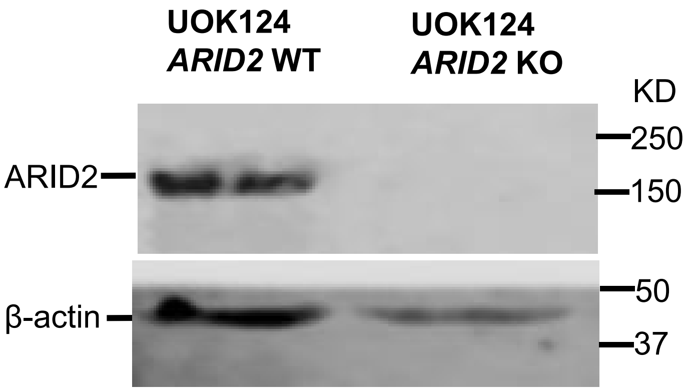

Figure S1 The Western blot (WB) analysis demonstrates that the UOK124 ARID2 WT cell line expresses ARID2, while the UOK124 ARID2 KO cell line shows a complete absence of ARID2 expression, confirming the successful generation of the ARID2 knockout cell line.

Supplemental Figure S2

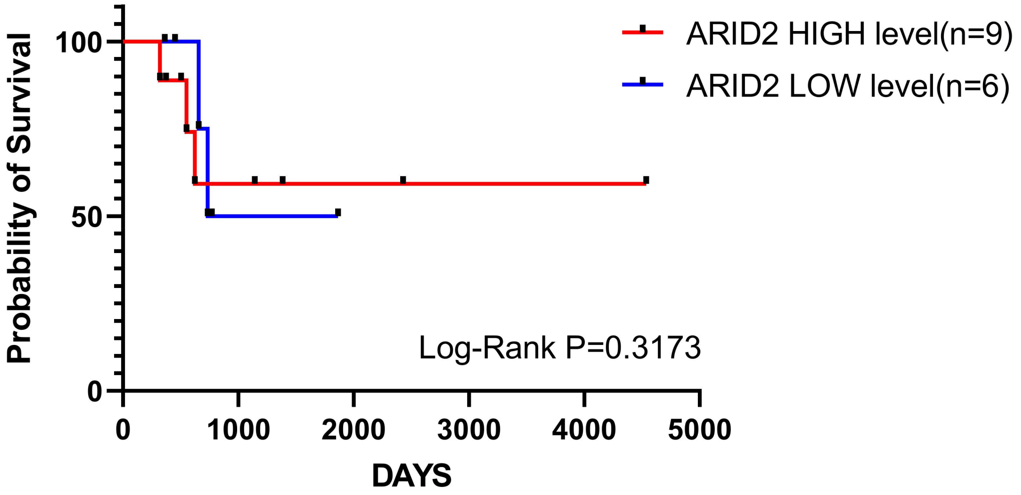

Figure S2 Kaplan-Meier analysis of overall survival for TFE3-RCC patients with HIGH and LOW ARID2 mRNA expressions in TCGA cohorts. p values were calculated using Log-Rank test.
